# The Emerging Role of CACNA1E in Cancer: Molecular Mechanisms and Therapeutic Implications

**DOI:** 10.7150/jca.133409

**Published:** 2026-06-04

**Authors:** Wei Hou

**Affiliations:** Translational Medicine Research Center, School of Clinical Medicine, Qilu Medical University, 1678 Renmin West Road, Zibo 255300, China.

**Keywords:** CACNA1E, voltage-gated calcium channels (VGCCs), cancer, prognostic biomarker, therapeutic target, chemoresistance

## Abstract

Voltage-gated calcium channels (VGCCs) are crucial membrane proteins that mediate calcium influx. The CACNA gene family, which encodes the alpha subunits of VGCC complexes, is essential for forming functional calcium channels and plays significant roles in various cancers. This review focuses on the CACNA1E gene, which encodes the Cav2.3 channel α1E subunit, and systematically elaborates its role in cancer. Studies have identified CACNA1E as a key prognostic stemness-related gene in bladder cancer. Its somatic mutations are associated with the development of air pollution-related lung cancer and non-small cell lung cancer. In breast cancer, genetic variations and DNA methylation differences in CACNA1E have also been reported. Functionally, CACNA1E drives tumor progression by regulating calcium signaling. For example, in osteosarcoma, METTL3-mediated m⁶A modification stabilizes CACNA1E mRNA, activating the WNT7B/Ca²⁺ signaling pathway and thereby promoting tumor progression and chemoresistance.

The expression of CACNA1E is closely linked to patient prognosis, with its amplification and overexpression correlating with relapse in favorable histology Wilms' tumor. Additionally, CACNA1E is involved in therapy resistance, as evidenced by its downregulation being associated with temozolomide resistance in glioblastoma. Collectively, this evidence suggests that CACNA1E, along with other VGCC members, may serve as a potential prognostic biomarker and therapeutic target in cancer. Future research should further explore their molecular mechanisms, interactions with the tumor microenvironment, and clinical translation potential.

## 1. Introduction

Voltage-gated calcium channels (VGCCs) are transmembrane protein complexes that serve as critical conduits for calcium ion (Ca²⁺) influx into cells upon membrane depolarization, thereby playing a fundamental role in converting electrical signals into intracellular biochemical events. These channels function as multi-subunit complexes, with the pore-forming α1 subunit—encoded by the CACNA gene family—determining the channel's core biophysical and pharmacological properties. The diversity of VGCC subtypes arises from multiple CACNA1 genes (including CACNA1A, 1B, 1C, 1D, 1E, 1F, and 1S), which exhibit tissue-specific expression patterns and are finely regulated at both transcriptional and post-translational levels. Auxiliary subunits (δ, α2/δ, and γ) further modulate channel trafficking and activity, adding additional layers of functional regulation.

Within this family, the CACNA1E (Calcium Voltage-Gated Channel Subunit Alpha1 E) gene encodes the α1E subunit (Cav2.3), which forms the principal component of R-type calcium channels 1,2. This channel subtype is predominantly expressed in the nervous system, with particularly high density in hippocampal presynaptic and postsynaptic regions. Within these neural contexts, Cav2.3 channels contribute to essential processes including neurotransmitter release and synaptic plasticity, such as long-term potentiation, which underlies learning and memory formation.

While the physiological role of CACNA1E in neuronal excitability is well-established, its function in non-excitable tissues and pathological contexts—particularly in oncology—represents an emerging and intriguing field of investigation. Calcium signaling operates as a double-edged sword in cellular homeostasis; its precise regulation is essential for normal function, whereas dysregulation has emerged as a hallmark of cancer, influencing fundamental processes including proliferation, migration, and apoptosis. Given that various VGCC family members have been implicated in tumor progression across different cancer types 3,4, the potential involvement of the neuronal channel CACNA1E in tumorigenesis poses a compelling scientific question worthy of systematic examination.

This review aims to synthesize the current evidence on the expression, regulation, and functional impact of CACNA1E across multiple cancer types 5-21, exploring its emerging role in tumor biology. By examining the diverse mechanisms through which CACNA1E contributes to cancer pathogenesis—including somatic mutations, epigenetic alterations, and dysregulated expression—we seek to evaluate its potential as a novel diagnostic marker and therapeutic target in oncology.

## 2. Functional Roles of CACNA1E in Various Cancers

### 2.1 Bladder Cancer

CACNA1E has been identified as a key prognostic stemness-related gene in bladder urothelial carcinoma, with a potential role in tumorigenesis, metastasis, and outcome prediction, particularly in the context of bone metastasis 5. Through multivariate Cox model and correlation analysis, it was identified as one of four key prognostic stemness-related genes alongside LINC01356, CGA, and SSX3. Furthermore, a set of 20 prognostic stemness-related genes was screened by Lasso regression, and based on them, a predictive model for the overall survival of BLCA (bladder urothelial carcinoma) patients was constructed, which achieved a relatively high accuracy with an area under the curve of 0.699.

Functionally, along with other key PSRGs, CACNA1E is part of a regulatory network linked to transcription factors (EPO and ARID3A) and correlates with hallmark cancer gene sets including DNA repair, Myc targets, E2F targets, mTORC1 signaling, and the unfolded protein response. This suggests its potential involvement in mediating BLCA bone metastatic progression.

The significance of CACNA1E is contextualized within the cancer stem cell framework. The mRNA expression-based stemness index was significantly upregulated in primary BLCA tissue and significantly correlated with poor patient prognosis, bone metastasis diagnosis, and advanced AJCC clinical stage. As a key prognostic stemness-related gene identified in this context, the role of CACNA1E aligns with the involvement of cancer stem cell characteristics in BLCA tumorigenesis and bone metastasis. In addition to the stemness-related role in BLCA, single-cell RNA sequencing of bladder squamous cell carcinoma (SCCB) revealed CACNA1E as one of the most variably expressed genes 6, suggesting its potential involvement in intratumoral heterogeneity and tumor evolution.

Collectively, these findings position CACNA1E as a component of stemness-related pathways that may influence BLCA aggressiveness and metastatic dissemination to bone, and further suggest its broader role in intratumoral heterogeneity in bladder cancer.

### 2.2 Breast Cancer

Beyond its role in bladder cancer, emerging genomic evidence has established CACNA1E as a recurrently mutated driver gene in breast cancer pathogenesis 7-12. In a whole-exome sequencing study of triple-negative breast cancer (TNBC) patients from the Mizo population in India, somatic mutation analysis revealed CACNA1E as one of the most frequently mutated genes, ranking among the top 20 most frequently mutated genes alongside TP53, IGSF3, RYR1, and FAM155A 7. The study suggests that CACNA1E may influence calcium signaling pathways, thereby affecting cell proliferation and metabolism. Supporting this finding, a targeted sequencing study in young breast cancer patients identified CACNA1E variants in both TNBC and luminal subtypes, suggesting it is probably a cancer driver in breast cancer for young women 8. Furthermore, research on early-onset luminal breast cancer also recognized its somatic mutations as potential cancer drivers 9. Together, these findings indicate that CACNA1E mutations are recurrently observed in both triple-negative and luminal breast cancers, suggesting a potential driver role that may extend across multiple subtypes. However, further studies are needed to extend these findings to other molecular subtypes and populations.

Beyond genetic alterations, dysregulation of CACNA1E expression appears to be involved in breast cancer development. A meta-analysis of public microarray datasets revealed that five VGCC family members—CACNA1A, CACNA1B, CACNA1E, CACNA1G, and CACNA1I—were under-expressed in breast cancer tissues compared to normal breast tissue, with a gene ranking in the top 1-10% of the low-expressed genes. This downregulation suggests a potential tumor-suppressive role for these calcium channels, including CACNA1E, in breast cancer pathogenesis 10. A methylation profiling study by Krasnyi et al. demonstrated that CACNA1E DNA methylation levels are significantly higher in small breast cancer tissues compared to both healthy breast tissue and fibroadenoma, with statistical significance in both test and validation cohorts (p < 0.001) 11. Furthermore, CACNA1E methylation correlated with tumor size and Luminal B subtype, and was incorporated into a four-gene diagnostic panel achieving an AUC of 0.99 for distinguishing malignant from benign lesions, suggesting this differential methylation could serve as a potential diagnostic biomarker for distinguishing malignant from benign lesions 11. In metastatic breast cancer, CACNA1E was identified as a candidate driver gene by an ensemble machine learning algorithm integrating mutation data from 450 metastatic samples 12. Pathway enrichment analysis (PEA) using ReactomeFIVIz (FDR < 0.03) for the top genes predicted by the EARN ensemble classifier revealed that CACNA1E is significantly enriched in the Neuronal System pathway—a metastasis-associated pathway specific to metastatic breast cancer (MBCA)—alongside genes such as GRIN1 and PLCB1 12. Additionally, CACNA1E participates in the Signal Transduction pathway, which is commonly enriched in both primary breast invasive carcinoma (BRCA) and metastatic breast cancer. Given its known function as a voltage-gated calcium channel, these findings suggest that CACNA1E may contribute to metastatic progression by engaging calcium signaling cascades within neuronal-like transcriptional programs, a mechanism consistent with its established roles in other cancer types.

In conclusion, current evidence indicates that CACNA1E contributes to breast cancer pathogenesis through multiple mechanisms, including somatic driver mutations, transcriptional downregulation, and epigenetic regulation. Its involvement across subtypes and disease stages highlights CACNA1E as a novel target with potential diagnostic and prognostic significance in breast cancer.

### 2.3 Gastric Cancer

CACNA1E also plays a role in gastric cancer by serving as a marker for a specific molecular subtype. Li et al. 13 performed a bioinformatics analysis of gastric cancer (GC) expression profiles and classified primary gastric adenocarcinomas into four molecular subtypes. CACNA1E was identified as a specific gene for Subtype 1, and a subtype-specific regulatory axis was proposed: the miR-202/CACNA1E/type II diabetes mellitus pathway. Notably, Subtype 1 also exhibited a higher rate of Helicobacter pylori infection compared to other subtypes, suggesting a potential link between infection, metabolic pathways, and CACNA1E expression 13.

However, it is important to note that this study was solely based on computational predictions without experimental validation. The proposed miR-202/CACNA1E interaction, the functional role of CACNA1E in GC cells, and the mechanistic connection to the type II diabetes mellitus pathway remain to be experimentally verified. Notably, the finding that MALAT1 drives gastric cancer progression by negatively regulating miR-202 14 raises the possibility of a broader MALAT1/miR-202/CACNA1E regulatory axis, a hypothesis that requires future experimental validation. Additionally, the clinical significance of CACNA1E in GC—such as its association with patient prognosis, chemoresistance, or therapeutic response—has not yet been explored.

Therefore, while CACNA1E may serve as a potential feature gene for identifying a distinct GC subtype, further functional and clinical studies are warranted to elucidate its precise role in gastric carcinogenesis and to evaluate its potential as a diagnostic biomarker or therapeutic target in this cancer type.

### 2.4 Glioblastoma (GBM)

CACNA1E has been implicated in glioblastoma (GBM) pathogenesis through multiple lines of evidence, encompassing both its role in therapeutic resistance and its potential as a prognostic biomarker. Nayak and Mallick 15 identified CACNA1E as a downregulated hub gene in temozolomide (TMZ)-resistant glioblastoma (GBM) cells. This downregulation was further associated with reduced expression of the long non-coding RNA RUSC2, which is predicted to post-transcriptionally regulate CACNA1E through the competing endogenous RNA (ceRNA) mechanism. Given that CACNA1E is involved in calcium signaling—a pathway critically implicated in the regulation of autophagy—the observed downregulation of CACNA1E may consequently contribute to TMZ resistance in GBM 15.

Independent bioinformatics analyses by Li et al. 16 integrated three GEO datasets (GSE90604, GSE50161, GSE134470) with TCGA data and identified CACNA1E as a significantly downregulated hub gene in GBM, with expression levels dropping from 21.65 TPM in normal brain to 0.8 TPM in tumor tissues. Notably, CACNA1E expression showed molecular subtype specificity, with upregulation in the mesenchymal subtype and downregulation in the proneural subtype, and a hazard ratio of 1.01 indicated an association with aggressive disease 16.

Collectively, these complementary studies position CACNA1E as both a mediator of chemotherapy resistance (via ceRNA-mediated downregulation) and a potential prognostic biomarker with subtype-specific expression patterns in GBM.

### 2.5 Non-Small Cell Lung Cancer (NSCLC)

CACNA1E has been identified as playing a significant pro-tumorigenic role in non-small cell lung cancer (NSCLC). Evidence for this role emerges from epidemiological, genomic, and functional studies 17,18. Notably, CACNA1E is among the genes exhibiting a significantly elevated mutation frequency in lung cancers linked to environmental carcinogen exposure 17. A genomic analysis of NSCLC patients from Xuanwei, China—a region characterized by severe air pollution from smoky coal combustion and a high incidence of lung cancer—revealed a distinct mutational profile compared to patients from regions where smoky coal was not used, with CACNA1E identified as one of the frequently mutated genes 17. Furthermore, the mutation rates of 70 genes, including CACNA1E, showed a positive correlation with patients' lifetime exposure to benzo(a)pyrene, a potent carcinogen present in coal smoke 17.

Beyond populations with high household air pollution exposure, mutations and overexpression of CACNA1E are prevalent in broader NSCLC cohorts and are associated with adverse clinical outcomes. Analysis of The Cancer Genome Atlas (TCGA) datasets indicates that CACNA1E is mutated in 12.8% of 1616 NSCLC patients 18. Consistently, both mRNA and protein expression levels of CACNA1E are elevated in NSCLC tumor tissues compared to adjacent non-tumoral tissues, and this high expression level is inversely correlated with patient prognosis, underscoring its clinical relevance.

The functional mechanisms underlying CACNA1E's oncogenic role involve the activation of calcium signaling and downstream growth factor pathways. Experimental studies demonstrate that overexpression of either wild-type or specific mutant variants (e.g., A275S or R249G) of CACNA1E promotes NSCLC cell proliferation and activates the epidermal growth factor receptor (EGFR) signaling pathway. Conversely, knockdown of CACNA1E exerts inhibitory effects on NSCLC cells. Mechanistically, CACNA1E enhances calcium channel current density and Ca²⁺ influx, and the proliferation of NSCLC cells can be suppressed by calcium channel blockers, confirming the functional dependence on calcium signaling 18.

In summary, these findings elucidate an important role for CACNA1E in the development and progression of NSCLC, particularly in cancers associated with environmental carcinogen exposure 17. Furthermore, evidence from broader NSCLC cohorts suggests that CACNA1E represents a potential therapeutic target, and its blockade may offer a novel strategy for NSCLC treatment 18.

### 2.6 Osteosarcoma

Further emphasizing the diverse mechanisms through which CACNA1E exerts its oncogenic effects, the gene is significantly upregulated in osteosarcoma tissues compared to adjacent normal tissues. This elevated expression is critically regulated at the post-transcriptional level by N6-methyladenosine (m6A) modification. Specifically, the m6A methyltransferase METTL3 acts as a "writer" to induce m6A hypermethylation on CACNA1E mRNA, thereby enhancing its stability and expression. Subsequently, the m6A "reader" protein IGF2BP2 recognizes and further stabilizes this modified transcript, amplifying the oncogenic signal 19.

Functional studies demonstrate that CACNA1E is a key driver of osteosarcoma malignancy. Knockdown of CACNA1E effectively inhibits both the growth and lung metastasis of osteosarcoma cells. Furthermore, high CACNA1E expression is clinically relevant as it is associated with resistance to methotrexate (MTX), a cornerstone chemotherapeutic agent for osteosarcoma. Consequently, suppressing CACNA1E can attenuate tumor cell resistance to MTX.

**Figure 1:**
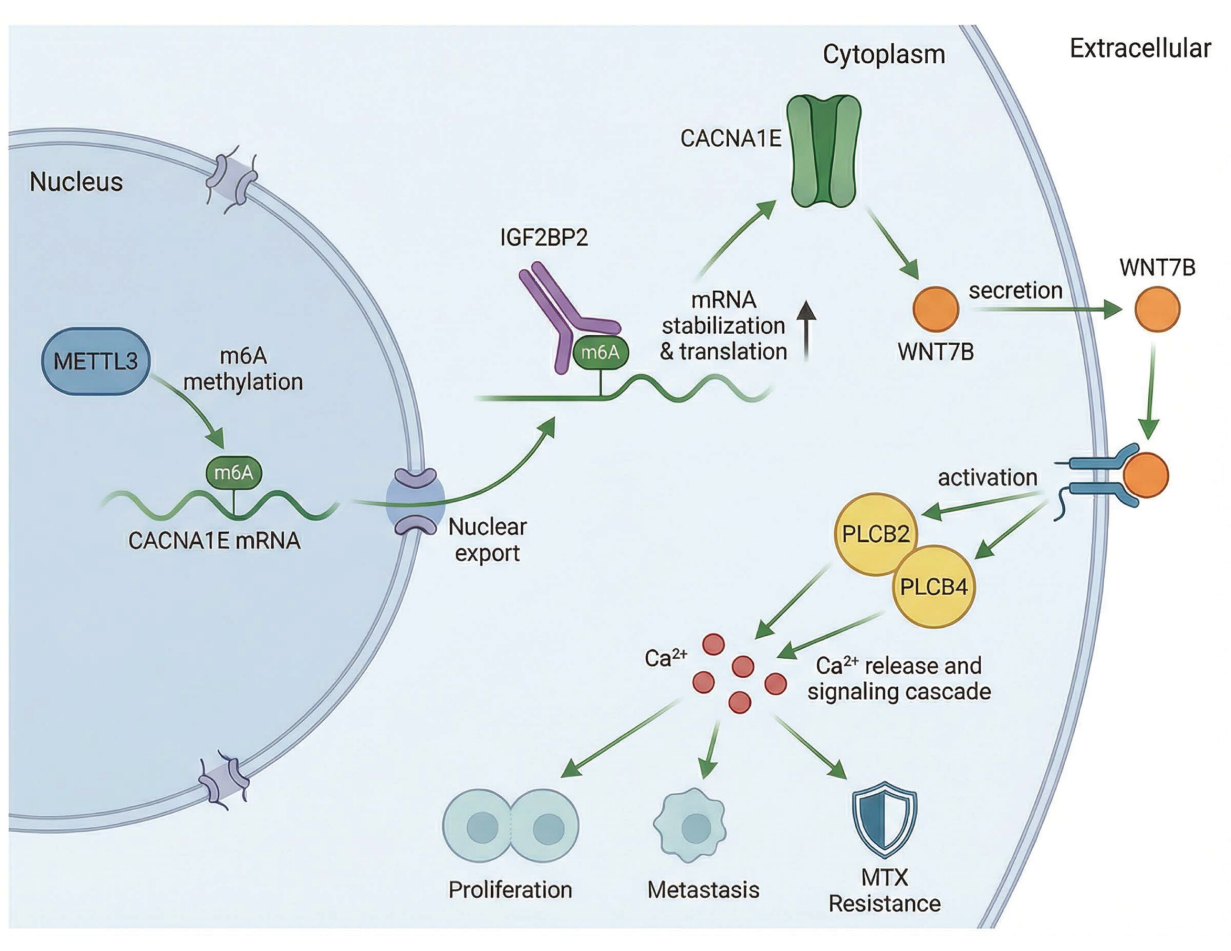
Mechanistic Role of CACNA1E Involved in Osteosarcoma.

The oncogenic mechanism of CACNA1E is mediated through the activation of the WNT7B/Ca²⁺ signaling pathway 19. CACNA1E transcriptionally upregulates WNT7B, which in turn enhances non-canonical Wnt/Ca²⁺ signaling. This cascade ultimately promotes osteosarcoma progression and confers MTX resistance, establishing a complete pro-tumorigenic axis wherein METTL3-mediated m6A modification of CACNA1E amplifies WNT7B-driven Ca²⁺ signaling (Figure 1).

Given that miR-342-5p has been identified as an upstream negative regulator of WNT7B in osteosarcoma 20, and that CACNA1E transcriptionally upregulates WNT7B to activate Ca²⁺ signaling 19, the potential interplay or convergence of these two distinct regulatory axes—the miR-342-5p/WNT7B and CACNA1E/WNT7B pathways—in modulating osteosarcoma progression and chemoresistance warrants further investigation.

These findings highlight significant therapeutic implications. A promising strategy involves the targeted inhibition of CACNA1E in combination with MTX chemotherapy. Experimental evidence indicates that this combination exerts a synergistic effect in suppressing osteosarcoma progression, offering a potential novel approach to overcome MTX resistance.

In summary, CACNA1E is an epigenetically regulated oncogene in osteosarcoma that promotes tumor growth, metastasis, and chemoresistance by activating the WNT7B/Ca²⁺ signaling pathway, positioning CACNA1E as a compelling therapeutic target, particularly in strategies aimed at reversing chemotherapy resistance.

### 2.7 Wilms' Tumor

The clinical significance of CACNA1E extends to pediatric malignancies as well, with the gene playing a critical role in Wilms' tumor relapse and poor prognosis through its amplification and overexpression. Studies identified a micro-amplification encompassing the CACNA1E gene at chromosome 1q25.3 in a subset of favorable histology Wilms' tumors, which correlated with significantly shorter relapse-free survival 21. At the DNA level, microamplification of CACNA1E was associated with an increased rate and shorter time to relapse. At the RNA level, CACNA1E mRNA overexpression was significantly associated with increased DNA copy number and was observed at higher levels in relapsing compared to non-relapsing tumors 21. At the protein level, immunohistochemistry against the CaV2.3 protein revealed aberrant nuclear localization in tumor cells, particularly in blastemal cells, which correlated with reduced relapse-free survival, especially in cases treated with preoperative chemotherapy 21. Mechanistically, stable overexpression of CaV2.3 in HEK293 cells led to activation of the MEK/ERK5/Nur77 signaling pathway and specific upregulation of immediate early response genes such as EGR1, EGR2, EGR3, FOS, and FOSB 21. Notably, upregulation of various Frizzled genes was also observed in CaV2.3-overexpressing cells, hinting at a potential role for Wnt/Ca²⁺ signaling in Wilms' tumor pathogenesis 21. These data identify CACNA1E and its protein product CaV2.3 as a unique genetic aberration with direct clinical relevance in Wilms' tumor relapse, suggesting a potential novel therapeutic target for relapsed patients. Future studies should evaluate the therapeutic potential of CaV2.3 channel blockers, such as SNX-482 or carbonic anhydrase inhibitors, in preclinical Wilms' tumor models 21.

## 3. Therapeutic Strategies Targeting CACNA1E

The emerging understanding of CACNA1E as a driver of tumor progression and chemoresistance has prompted investigations into its therapeutic potential. Although no CACNA1E-targeted agents have yet entered clinical trials, preclinical studies have identified two promising strategies.

### 3.1. Drug Repurposing with Calcium Channel Blockers

In non-small cell lung cancer (NSCLC), Gao et al. 18 demonstrated that dihydropyridine calcium channel blockers—lercanidipine hydrochloride and nitrendipine—as well as the Cav2.3/R-type calcium-current inhibitor lamotrigine, significantly suppressed proliferation and colony formation of NSCLC cell lines. Mechanistically, knockdown of CACNA1E reduced intracellular calcium concentration in NSCLC cells and attenuated activation of the epidermal growth factor receptor (EGFR) signaling pathway, evidenced by decreased levels of phosphorylated EGFR (pEGFR), pAKT, and pERK. The observed anti-NSCLC activity of existing cardiovascular calcium channel blockers thus provides a rationale for future research exploring the repurposing of these agents for lung cancer therapy.

### 3.2. Targeted Inhibition Combined with Chemotherapy

In osteosarcoma (OS), Chen et al. 19 showed that CACNA1E is markedly upregulated in methotrexate (MTX)-resistant cells and that genetic silencing of CACNA1E restores MTX sensitivity. Importantly, the combination of CACNA1E knockdown and MTX treatment exerted a synergistic antitumor effect in both *in vitro* and *in vivo* models, effectively overcoming MTX resistance. Mechanistically, this synergy is mediated through the WNT7B/Ca²⁺ signaling axis 19.

These preclinical findings collectively position CACNA1E as a promising therapeutic target, providing a rationale for developing CACNA1E-targeted strategies in combination with standard chemotherapy.

### 3.3 Limitations and Challenges in Targeting CACNA1E

Despite the promising preclinical evidence, several significant challenges must be addressed before CACNA1E can be translated into clinical practice.

First, although Gao et al. 18 demonstrated anti-NSCLC activity of lercanidipine, nitrendipine, and lamotrigine, these agents are not selective for CACNA1E, underscoring the urgent need for selective CACNA1E antagonists.

Second, the calcium channel Cav2.3, encoded by CACNA1E, is involved in presynaptic calcium signaling that facilitates neurotransmitter release and contributes to short-term synaptic plasticity. Given the broad expression of calcium channels in multiple organ systems and the association of CACNA1E variants with severe neurodevelopmental phenotypes, hypothetical concerns exist that systemic inhibition of CACNA1E could potentially lead to adverse neurological effects.

Third, CACNA1E exhibits context-dependent roles: it acts as an oncogene in non-small cell lung cancer and osteosarcoma 18,19 but is downregulated in temozolomide-resistant glioblastoma 15. This duality poses a fundamental challenge for therapeutic generalization.

Fourth, future drug discovery efforts should employ structure-based drug design or high-throughput screening targeting the Cav2.3 channel to overcome the lack of selective CACNA1E antagonists.

Fifth, given the context-dependent dual role of CACNA1E, precision patient stratification will be critical; prospective validation of predictive biomarkers will help identify patient subsets most likely to benefit from CACNA1E-targeted inhibition.

Addressing these limitations will require concerted efforts in medicinal chemistry, mechanistic pharmacology, and clinically relevant preclinical modeling to advance CACNA1E-targeted strategies.

## 4. Conclusion and Outlook

**Figure 2:**
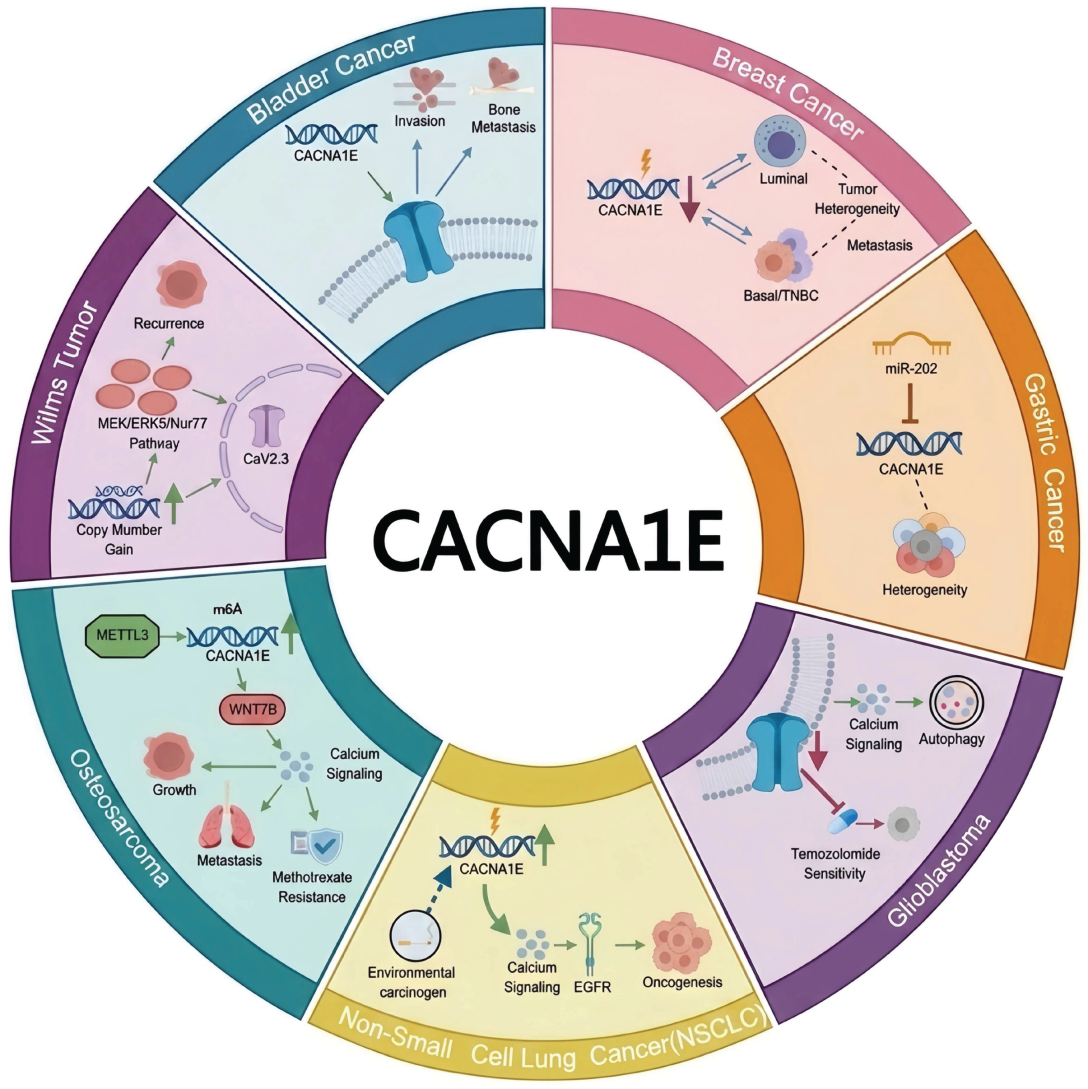
Roles of CACNA1E in Various Cancers.

The CACNA1E gene, which encodes the α1 subunit of the R-type calcium channel, plays a significant role in tumorigenesis and cancer progression. In this review, we summarize the mutations, expression patterns, and functional implications of CACNA1E across various cancer types (Figure 2). In breast cancer, genomic evidence has established CACNA1E as a recurrently mutated driver gene, with whole-exome sequencing of triple-negative breast cancer (TNBC) patients from the Mizo population in India identifying CACNA1E as one of the most frequently somatically mutated genes 7. Furthermore, targeted sequencing in young breast cancer patients (including TNBC and luminal subtypes) revealed CACNA1E variants, suggesting that CACNA1E is likely a cancer driver in young women 8. In non-small cell lung cancer (NSCLC), CACNA1E has been shown to play a significant pro-tumorigenic role 18, with genomic analysis uncovering a distinct mutational profile in which CACNA1E was frequently mutated 17. In glioblastoma (GBM), CACNA1E has been implicated in the development of temozolomide (TMZ) resistance 15. In Wilms' tumor, amplification and overexpression of CACNA1E play critical roles in tumor relapse and poor prognosis, suggesting CACNA1E as a potential novel therapeutic target for relapsed patients 21.

Beyond these cancer-type-specific observations, further progress in understanding CACNA1E in oncology depends on addressing four key areas: the gene's intrinsic molecular complexity arising from alternative splicing, its integration into broader regulatory networks such as the CDKL5 signaling axis, the unresolved mechanisms underlying its context-dependent functions in the tumor microenvironment and chemoresistance, and ultimately, the translation of these insights into precision therapeutic strategies.

### 4.1 Splice Variants and Functional Diversity

Adding another layer of complexity, numerous splice variants of CACNA1E have been identified, highlighting the gene's complexity and potential functional diversity 22-27. For instance, long-read RNA sequencing has been used to catalog splice variants of CACNA1E, resulting in the identification of 2,110 transcripts, of which up to 154 are predicted to encode functional channels based on their amino acid sequences 27. This foundational work sets the stage for future investigations into how specific splice variants influence channel function and contribute to disease states.

### 4.2 CDKL5 and CACNA1E: Functional Interactions

Recent studies have also established that the brain-enriched kinase CDKL5 acts as a physiological kinase that phosphorylates the voltage-gated calcium channel Cav2.3, which is encoded by CACNA1E 28. This phosphorylation is critical for normal channel function; loss of CDKL5 activity results in slowed channel inactivation, heightened sensitivity to cholinergic stimulation, and consequently, pathologically elevated neuronal excitability. In cancer contexts, however, the tumor-promoting effects of CDKL5 appear to be independent of CACNA1E.

For example, in glioblastoma, CDKL5 was identified as a survival kinase gene essential for cell viability. Its protein expression is elevated in over 8% of glioma cases, and high CDKL5 expression correlates with significantly poorer prognosis, including shorter overall survival and increased mortality risk 29. Similarly, in breast cancer, the long non-coding RNA LINC00680 upregulates CDKL5 protein levels by sequestering miR-320b, thereby relieving its repression of CDKL5 mRNA and ultimately promoting docetaxel resistance 30. Moving forward, it will be important to systematically examine whether CDKL5 also phosphorylates and regulates Cav2.3 in cancer types where CDKL5 is known to be highly expressed—such as glioblastoma and breast cancer. If this regulation is absent, future studies should evaluate the therapeutic potential of selective Cav2.3 channel inhibitors in models exhibiting aberrant CDKL5 expression.

### 4.3 Unresolved Mechanisms Underlying CACNA1E Function

Looking ahead, future research should delve deeper into the specific molecular mechanisms of CACNA1E in tumors and its interaction with the tumor microenvironment. For instance, in osteosarcoma, CACNA1E promotes progression and chemoresistance by activating the WNT7B/Ca²⁺ signaling pathway, suggesting that similar or unique signaling axes may exist in other cancers and await discovery 19. Notably, the association between CACNA1E and chemoresistance appears context-dependent: while its high expression drives methotrexate resistance in osteosarcoma 19, bioinformatic analyses have suggested that its downregulation may be associated with temozolomide resistance in glioblastoma 15. Future studies need to systematically elucidate its mechanism of action in response to various therapeutic agents across different tumor types to overcome technical and interpretative challenges in clinical application.

Furthermore, the post-transcriptional regulatory mechanisms of CACNA1E warrant systematic investigation. These include the m⁶A modification that regulates its stability and expression, as documented in osteosarcoma 19. Future studies may also explore other potential regulatory layers, such as alternative splicing, DNA methylation, and ceRNA networks, which have been implicated in the regulation of CACNA1E or related calcium channels in other cancer types.

Another outstanding question concerns the functional interactions between CACNA1E and other calcium channel subunits (e.g., CACNA1C, CACNA1H, CACNA1G) 31-44, and whether they cooperatively regulate calcium flux and influence drug sensitivity, remain to be elucidated.

### 4.4 Toward CACNA1E-Targeted Precision Oncology

The emerging understanding of CACNA1E as a context-dependent driver of tumor progression and chemoresistance opens new avenues for precision oncology. To achieve clinical translation, future research should integrate multi-omics data and conduct large-scale clinical validation to confirm its value as a prognostic biomarker, as suggested by findings in non-small cell lung cancer, bladder cancer, glioblastoma, and Wilms' tumor. Based on current preclinical evidence, several therapeutic strategies merit prioritization.

First, given the limited selectivity of existing calcium channel blockers 18, developing highly selective CACNA1E inhibitors—either small molecules or monoclonal antibodies—represents an urgent priority. Structure-based drug design and high-throughput screening targeting the Cav2.3 channel pore domain or allosteric sites may accelerate this effort. Second, clinical success will depend on robust predictive biomarkers; candidates include CACNA1E overexpression 18,19, gain-of-function mutations (e.g., A275S, R249G) 18, and m6A modification status 19, warranting prospective validation in biomarker-enriched trials. Multi-omics integration—combining genomic, transcriptomic, epigenomic, and proteomic data—will be essential to refine these biomarker candidates and enable precise patient stratification.

Third, the synergy between CACNA1E inhibition and methotrexate in osteosarcoma 19, together with suppression of EGFR signaling upon CACNA1E knockdown in NSCLC 18, supports rational combination regimens with chemotherapies, or EGFR inhibitors.

Fourth, while CACNA1E inhibition appears beneficial in cancers where it acts as an oncogene (NSCLC, osteosarcoma, Wilms tumor), alternative strategies—such as epigenetic modulators (e.g., DNA methyltransferase inhibitors) or gene therapy approaches aimed at restoring CACNA1E expression—may be required for cancers exhibiting CACNA1E downregulation.

Finally, to improve translational fidelity, future efficacy studies should employ orthotopic tumor models that recapitulate the native tissue microenvironment, patient-derived xenografts (PDX), and immunocompetent systems to assess immune-mediated responses. Large-scale clinical validation across diverse cancer types and populations will be indispensable to confirm the prognostic and predictive value of CACNA1E.

Collectively, these strategies—ranging from multi-omics integration and biomarker discovery to selective inhibitor development and rational combination regimens—hold promise for translating CACNA1E-targeted approaches into clinical practice. The ultimate goal is to establish CACNA1E as both a diagnostic marker and a therapeutic target, thereby significantly advancing precision oncology for patients with CACNA1E-driven malignancies.

In conclusion, current evidence indicates that CACNA1E contributes to cancer pathogenesis through multiple mechanisms, including somatic driver mutations, transcriptional dysregulation, and epigenetic alterations. Its involvement across cancer subtypes and disease stages underscores CACNA1E as a novel target with potential diagnostic, prognostic, and therapeutic significance.

## Abbreviations

VGCCs: voltage-gated calcium channels
CACNA1E: calcium voltage-gated channel subunit alpha1 E
CDKL5: cyclin-dependent kinase-like 5
EMT: epithelial-mesenchymal transition
NSCLC: non-small cell lung cancer
ceRNA: competing endogenous RNA
BLCA: bladder urothelial carcinoma
TNBC: triple-negative breast cancer
BRCA: breast invasive carcinoma
GBM: glioblastoma
TMZ: temozolomide
TCGA: The Cancer Genome Atlas
EGFR: epidermal growth factor receptor
m6A: N6-methyladenosine
METTL3: methyltransferase-like 3
IGF2BP2: insulin-like growth factor 2 mRNA-binding protein 2
MTX: methotrexate
OS: osteosarcoma
PDX: patient-derived xenografts
